# Childhood conduct disorder trajectories, prior risk factors and cannabis use at age 16: birth cohort study

**DOI:** 10.1111/add.12268

**Published:** 2013-07-12

**Authors:** Jon Heron, Edward D Barker, Carol Joinson, Glyn Lewis, Matthew Hickman, Marcus Munafò, John Macleod

**Affiliations:** 1 School of Social and Community Medicine, University of BristolBristol, UK; 2 Department of Psychological Sciences, Birkbeck, University of LondonLondon, UK; 3 School of Experimental Psychology, University of BristolBristol, UK

**Keywords:** Adolescence, ALSPAC, cannabis problems, cannabis use, conduct disorder, risk factors, trajectories

## Abstract

**Aims**To investigate the prevalence of cannabis use and problem use in boys and girls at age 16 years, and to investigate the role of adversity in early life and of conduct disorder between the ages of 4 and 13 years as risk factors for these outcomes.

**Design**Birth cohort study.

**Setting**England.

**Participants**A total of 4159 (2393 girls) participants in the Avon Longitudinal Study of Parents and Children (ALSPAC) birth cohort providing information on cannabis use at age 16.

**Measurements**Cannabis use and problem cannabis use at age 16 were assessed by postal questionnaire. Material adversity, maternal substance use, maternal mental health and child conduct disorder were all assessed by maternal report.

**Findings**Cannabis use was more common among girls than boys (21.4% versus 18.3%, *P* = 0.005). Problem cannabis use was more common in boys than girls (3.6% versus 2.8%, *P* = 0.007). Early-onset persistent conduct problems were associated strongly with problem cannabis use [odds ratio (OR) = 6.46, 95% confidence interval (CI) = 4.06–10.28]. Residence in subsidized housing (OR = 3.10, 95% CI = 1.95, 4.92); maternal cannabis use (OR 8.84, 95% CI 5.64–13.9) and any maternal smoking in the postnatal period (OR = 2.69, 95% CI = 1.90–3.81) all predicted problem cannabis use. Attributable risks for adolescent problem cannabis use associated with the above factors were 25, 13, 17 and 24%, respectively.

**Conclusions**Maternal smoking and cannabis use, early material disadvantage and early-onset persistent conduct problems are important risk factors for adolescent problem cannabis use. This may have implications for prevention.

## Introduction

Cannabis use among young people is common, with peak incidence of onset of use occurring in adolescence 1–2. Use is associated with many adverse health and social outcomes, with these associations being most clearly apparent in relation to more extreme cannabis use phenotypes. This could simply reflect a dose–response relation between cannabis use and harm; however, it might also suggest a distinct phenotype of problem cannabis use whose effects, and possibly antecedents, are different. Instruments to measure problem cannabis use have been developed to allow investigation of these questions 3.

Several longitudinal studies have investigated associations between conduct disorder and both cannabis use and problem cannabis use 4–8. A relatively consistent association between childhood conduct disorder and both these outcomes has been apparent in these investigations 9. Recent evidence suggests that conduct disorder is, itself, not a single phenomenon but rather comprises four distinct longitudinal phenotypes apparent between the ages of 4 and 13 years 10. Outcomes of conduct disorder might vary according to these phenotypes; however, this question has received limited attention with regard to subsequent cannabis use 4. Risk factors for conduct disorder, particularly early-onset persistent conduct disorder, have been found previously to include parental substance use and mental health problems, early-life social disadvantage and family adversity and childhood victimization 11. Adolescent cannabis use has also been shown to be associated with these early-life factors 12–13. This association could arise because these factors predispose to conduct disorder which, in turn, predisposes to substance use, or it might arise through other pathways including both other environmental influences and genetic factors shared between parents and offspring 12–13.

In a large UK birth cohort we examined associations of conduct disorder trajectories between the ages of 4 and 13 years with cannabis use at age 16. We also examined the associations of parental substance use and mental health problems, early material disadvantage and early family adversity and subsequent conduct problem trajectories with cannabis use at age 16.

## Methods

### Participants

The sample comprised participants from the Avon Longitudinal Study of Parents and Children (ALSPAC) 14,15. Pregnant women resident in the former Avon Health Authority (Bristol) in South West England with an estimated date of delivery between 1 April 1991 and 31 December 1992 were invited to take part, resulting in a ‘core’ cohort of 14 541 pregnancies and 13 617 singletons alive at 12 months of age. Within this sample, 7218 singleton individuals had information allowing the derivation of conduct problem trajectory. Of these individuals, 4159 (57.6%) had information on cannabis use at age 16, 2393 of these individuals were girls (4822 individuals in total provided information on cannabis use at 16). The primary source of data collection was via self-completion questionnaires administered at least annually to the mother, her partner and the ALSPAC study child. Since the age of 7 years the whole offspring cohort has been invited to annual ‘focus’ clinic for a variety of hands-on assessments. More detailed information on the ALSPAC study is available at http://www.alspac.bris.ac.uk. All aspects of the study were reviewed and approved by the ALSPAC Law and Ethics Committee, which is registered as an Institutional Review Board. Approval was also obtained from the National Health Service Local Research Ethics Committees.

### Measures used

A range of risk factors from early life measured prior to conduct problems were considered. These were derived from a number of questionnaires administered to the young person's parents between enrolment (early in antenatal period) and 47 months after the birth (i.e. up until the first measure of conduct problems). The choice of variables was parsimonious and was guided by considerations around the best use of available information on the possible influences discussed above, data completeness and collinearity. Previous work in ALSPAC found family position to be associated with adolescent alcohol use 17. Other than sex and family position (coded as whether study child is first/second/third child or greater) early-life risk factors were considered in the following categories.

#### Social position and family adversity

Housing tenure (coded as owned/mortgaged, privately rented, subsidized housing rented from council/housing association), crowding status (coded as a value greater than 1 for the ratio of number of residents to number of rooms in house), maternal educational attainment (coded as no high school qualifications, high school, beyond high school), quintiles of household disposable income [a measure assessing income when the child was a toddler (2–4 years of age)] and accounting for family size and composition and estimated housing benefits 18 and finally parental social class (the highest social class of either parent) at enrolment based on the Registrar General's classification of occupations: I/II (professional/managerial and technical) versus IIINM or lower (skilled non-manual/-manual, semiskilled and unskilled).

#### Maternal substance use and depressive symptoms

Maternal postnatal cannabis use (‘yes’ indicating a positive response at either 2, 8, 21 or 33 months postnatal), maternal daily alcohol use (‘yes’ indicating a positive response at either 2, 8, 21 or 33 months postnatal), maternal smoking (none/one to 19 cigarettes per day/20+ per day, indicating the greatest amount reported at either 2, 8, 21 or 33 months postnatal), maternal postnatal depressive symptoms [achieving a score of 13 or higher on the Edinburgh Postnatal Depression Scale (EPDS) 19 at either 2, 8, 21 or 33 months postnatal] and antenatal maternal depressive symptoms (achieving a score of 13 or higher on the EPDS at either 18 or 32 weeks gestation). Additional data were also available on the substance use behaviour of the mother's partner. These data were used in preliminary analyses but were not taken forward to the final multivariable models due to poorer response rates, strong collinearity with the maternal measures, similarity in associations (compared with the maternal measures) and issues with imputing this information when the young person has only one parent.

Measures of childhood victimization were not included in these analyses. Only 12 cases of sexual victimization prior to age 4 years were reported by mothers in ALSPAC. Some data were available on whether or not children appeared on the child protection register in early life; however, more than 90% of such children had no record of follow-up beyond the point of their appearance on the register.

#### Conduct problem trajectories

The derivation of trajectories of conduct problems has been reported previously 10. Briefly, latent class growth analysis models fitted in Mplus 20 were applied to six binary indicators of conduct problems derived from the ‘conduct problem’ scale of the Strengths and Difficulties Questionnaire (SDQ 21–22), which was dichotomized at the threshold 4/5 21. The six repeated measures spanned the age period from 47 months to 13 years. The four resulting trajectories were described as low (64.3%), childhood-limited (14.7%), adolescent-onset (11.8%) and early-onset persistent (9.2%).

#### Cannabis use and problem use

Information on cannabis use was collected via a postal questionnaire that the young people completed at age 16 years [median age 16 years 7 months, interquartile range (IQR) = 16 years 6 months–16 years 10 months]. Respondents completed the six-item Cannabis Abuse Screen Test (CAST 23), along with an initial stem-question asking whether the respondent has used cannabis since the age of 15. Items in the CAST relate to morning cannabis use, use while alone, experience of cannabis-related problems and failed attempts to quit or reduce use. The CAST sum-score was derived as illustrated by Piontek and colleagues 3, where a response to each item of fairly often/very often is coded as one and never/rarely as zero. A three-level ordered categorical variable was derived indicating: 0, no use since age 15; 1, yes to use but a CAST sum-score of zero; and 2, yes to use and a non-zero CAST sum-score. While a cut-point of 4 has been recommended 3, we opted for a cut of 1 or more points in this sample of young adolescents in order to yield an adequate number of respondents in the highest group. We defined this cannabis outcome as 0 ‘no use’, 1 ‘cannabis use’ and 2 ‘problem cannabis use’.

### Statistical methods

#### Modelling strategy

A set of multinomial regression models were performed using the measure of cannabis use/problem use as a three-category outcome. First, the univariate effect of each early-life predictor on this cannabis measure was assessed followed by adjustment for conduct problem trajectory. Secondly, the univariate effect of conduct problem trajectory on cannabis use/problem use was assessed, followed by adjustment for the same set of early-life predictors. Finally, to evaluate the possible public health importance of these childhood influences on adolescent cannabis use we estimated the population attributable risk fraction (PARF) associated with the factors associated most strongly and substantially with cannabis problems 24. All regression analyses were carried out in Latent Gold version 4.5.0.11145 25. For all models containing the conduct problems measure, a ‘three-step’ approach was followed in order to utilize the variable derived previously 10 but to adjust for the potential impact of classification error in this variable (for detail see Appendix).

#### Multiple imputation

Multiple imputation was based on the 7218 individuals with information on the conduct–problem trajectory, the main exposure considered derived from those respondents who had four or more of the six SDQ measures required for the original mixture modelling 10. The preliminary multinomial regression models described above employed list-wise deletion, and hence sample sizes varied from the full sample of 7218 for gender to samples of down to 4000 for some of the less complete measures. Consequently, the complete case sample for which we could perform multivariable models was smaller than 7218. To address this problem, missing data imputation was carried out by chained equations 26 using the *ice* routine 27 in Stata to restore the sample size to 7218 for all analyses. The imputation model contained the conduct problem trajectory assignment, the other outcomes and risk factors described above, as well as a number of additional auxiliary variables known to be related both to missingness and to the key variables of interest in our models. One hundred data sets were imputed, and these data were exported to Latent Gold. Final model estimates were pooled using Rubin's rules 28.

## Results

### Prevalence of key measures

Of the 4159 individuals reporting cannabis use at age 16, 835 (20.1%) were classified as using cannabis without problem use and a further 130 (3.1%) reported problem use. Cannabis use without reported problems was more common among girls than boys (21.4% versus 18.3%, *P* = 0.005). Problem use was more common among boys than girls (3.65% versus 2.8%, *P* = 0.007). The most frequently endorsed item from the CAST was ‘use leading to memory problems’, with 47% of problem users reporting that this occurred ‘fairly often’ or ‘often’. Table 1 shows the effect of imputation on the distribution of participants across categories of the variables utilized.

**Table 1:** Distribution of main variables (complete case and imputed data)

		Complete case (numbers vary)	Following imputation (n *=* 7218)
Conduct problems	Low	5061 (70.1%)	Data were complete in this sample
	Childhood limited	875 (12.1%)	
	Adolescent onset	616 (8.5%)	
	Early-onset persistent	666 (9.2%)	
Cannabis use	Non-use	3194 (76.8%)	76.3%
	Use	835 (20.1%)	19.7%
	Problem use	130 (3.1%)	4.0%
Housing tenure	Mortgaged/owned	5885 (83.4%)	83.2%
	Private rented	559 (7.9%)	8.0%
	Subsidized rented	617 (8.7%)	8.8%
Parity	First-born	3295 (46.8%)	46.8%
	Second-born	2511 (35.7%)	35.7%
	Third-born plus	1230 (17.5%)	17.5%
Home overcrowding	Up to 1 person/room	6724 (96.3%)	96.2%
	>1 person/room	262 (3.8%)	3.8%
Maternal education	A level or higher	3070 (43.4%)	43.2%
	O-level	2507 (35.5%)	35.5%
	< O-level	1490 (21.1%)	21.3%
Household income	Top 20%	1561 (23.0%)	22.9%
	Middle 60%	4155 (61.3%)	61.4%
	Lowest 20%	1060 (15.6%)	15.7%
Social class	Managerial/professional	4156 (61.5%)	60.5%
	III or lower	2606 (38.5%)	39.5%
Maternal cannabis	No	5624 (95.4%)	95.1%
	Some use postnatally	269 (4.6%)	4.9%
Maternal alcohol	Less than daily	5273 (83.5%)	83.6%
	Daily use	1043 (16.5%)	16.4%
Maternal smoking	No	4775 (77.8%)	76.1%
	Some use postnatally	1363 (22.2%)	23.9%
Antenatal maternal depressive symptoms	Subthreshold throughout period	5354 (82.4%)	81.8%
	EPDS > 12 at some point	1145 (17.6%)	18.2%
Postnatal maternal depressive symptoms	Subthreshold throughout period	4846 (79.3%)	78.1%
	EPDS > 12 at some point	1266 (20.7%)	21.9%

EPDS = Edinburgh Postnatal Depression Scale.

### Early-life predictors and cannabis use/problem use

Table 2 shows the association between early-life predictors and cannabis use/problem use. There was good agreement with those obtained using complete case data (see Supporting information, Table S1). Patterns of association between these exposures and any cannabis use were different from those seen with problem cannabis use. In general, indicators of greater early adversity showed a stronger and more substantial association with problem cannabis use rather than any cannabis use. Some indicators of increased adversity, such as lower maternal education, were actually associated with lower risk of any cannabis use. In general, associations between adversity and any cannabis use were small and estimated imprecisely, whereas those with problem cannabis use were of greater magnitude and stronger. The main exceptions to this pattern were seen in relation to maternal substance use—maternal tobacco, alcohol and cannabis use were all associated relatively strongly with both any use and problem use of cannabis by their offspring at age 16. The largest effect was seen with maternal cannabis use. For maternal cannabis use and maternal tobacco use the effect appeared greater on risk of problem cannabis use, whereas with maternal alcohol use the stronger and more substantial effect was on any use of cannabis. Risk of any cannabis use was higher among girls compared to boys, whereas the opposite was true for risk of problem cannabis use. Adjustment for conduct problem trajectory attenuated these associations although, in general, they remained strong and substantial.

**Table 2:** The effect of early-life predictors on cannabis use and problem use at age 16 (results from multiple imputation = 7218)

	Univariable associations (reference for outcome *=* no cannabis use)			Individually adjusted for conduct trajectory (reference for outcome *=* no cannabis use)		
	Cannabis use	Problem use	Omnibus P-value	Cannabis use	Problem use	Omnibus P-value
Gender (ref = male)						
Female	1.22 (1.05, 1.42)	0.73 (0.51, 1.03)	0.005	1.24 (1.07, 1.43)	0.77 (0.54, 1.10)	0.007
Housing tenure (ref = mortgaged/owned)						
Private rented	1.41 (1.09, 1.81)	1.71 (1.04, 2.82)	<0.001	1.37 (1.07, 1.77)	1.55 (0.93, 2.56)	<0.001
Subsidized rented	1.12 (0.81, 1.54)	3.10 (1.95, 4.92)		1.07 (0.78, 1.48)	2.52 (1.57, 4.06)	
Parity (ref = first born)						
Second-born	1.09 (0.93, 1.29)	1.32 (0.93, 1.88)	0.033	1.10 (0.93, 1.29)	1.34 (0.94, 1.91)	0.045
Third-born plus	1.25 (1.02, 1.55)	1.73 (1.08, 2.77)		1.26 (1.02, 1.55)	1.68 (1.05, 2.71)	
Home overcrowding (ref = up to 1 person/room)						
>1 person/room	0.90 (0.58, 1.41)	1.61 (0.76, 3.42)	0.374	0.87 (0.56, 1.36)	1.33 (0.61, 2.88)	0.605
Maternal education (ref = A-level or higher)						
O-level	0.70 (0.60, 0.82)	1.10 (0.78, 1.54)	<0.001	0.69 (0.59, 0.82)	1.04 (0.74, 1.47)	<0.001
<O-level	0.62 (0.50, 0.77)	1.34 (0.85, 2.11)		0.60 (0.49, 0.74)	1.16 (0.73, 1.84)	
Household income (ref = top 20%)						
Middle 60%	0.83 (0.70, 0.99)	1.15 (0.77, 1.73)	0.050	0.83 (0.70, 0.98)	1.12 (0.74, 1.68)	0.148
Lowest 20%	0.85 (0.67, 1.09)	1.71 (0.96, 3.03)		0.82 (0.64, 1.05)	1.42 (0.79, 2.56)	
Social class (ref = managerial/professional)						
III or lower	0.74 (0.62, 0.88)	1.40 (0.95, 2.08)	<0.001	0.73 (0.61, 0.86)	1.30 (0.87, 1.93)	0.001
Maternal cannabis (ref = no)						
Some use postnatally	3.56 (2.59, 4.90)	8.84 (5.64, 13.9)	<0.001	3.51 (2.55, 4.83)	8.15 (5.11, 13.0)	<0.001
Maternal alcohol (less than daily)						
Daily use	1.70 (1.41, 2.05)	1.33 (0.90, 1.99)	<0.001	1.72 (1.43, 2.07)	1.39 (0.92, 2.09)	<0.001
Maternal smoking (ref = no)						
Some use postnatally	1.59 (1.33, 1.90)	2.69 (1.90, 3.81)	<0.001	1.55 (1.30, 1.86)	2.34 (1.63, 3.35)	<0.001
Antenatal maternal depressive symptoms						
EPDS > 12 at some point	1.04 (0.83, 1.30)	1.41 (0.89, 2.22)	0.303	0.99 (0.79, 1.23)	1.13 (0.70, 1.80)	0.861
Postnatal maternal depressive symptoms						
EPDS > 12 at some point	1.09 (0.90, 1.32)	1.89 (1.29, 2.77)	0.003	1.03 (0.85, 1.25)	1.48 (0.99, 2.21)	0.136

Estimates shown are multinomial odds ratios complete with 95% confidence intervals. Estimates are accompanied by omnibus *P*-values derived from Wald tests (with 2 degrees of freedom). EPDS = Edinburgh Postnatal Depression Scale.

### Conduct problems and cannabis at age 16

Table 3 shows associations between conduct problem trajectories and cannabis use at 16. Risk of both any use of cannabis and of problem cannabis use is elevated among children with conduct problems. The effect on any cannabis use is generally small and estimated imprecisely, with the exception of the association between adolescent-onset conduct problems and any cannabis use. For all conduct problem categories the effect on problem cannabis use is greater than that on any cannabis use. This is particularly evident in relation to early-onset persistent conduct problems that are associated with a greater than sixfold increased risk of problem cannabis use. These associations are attenuated after adjustment for earlier life factors preceding conduct problems, although in the case of early-onset persistent conduct problems and problem cannabis use the effect remains strong and substantial.

**Table 3:** The effect of conduct problem trajectory on cannabis use and problem use at age 16

	Unadjusted		Adjusted^a^	
Conduct problems	Cannabis use	Problem use	Cannabis use	Problem use
Low (reference)				
Childhood limited	1.26 (0.86, 1.85)	1.30 (0.45, 3.77)	1.26 (0.84, 1.88)	1.09 (0.36, 3.25)
Adolescent onset	1.78 (1.18, 2.67)	2.43 (0.91, 6.48)	1.89 (1.24, 2.88)	2.55 (1.00, 6.50)
Early-onset persistent	1.40 (0.98, 2.01)	6.46 (4.06, 10.28)	1.42 (0.98, 2.07)	4.97 (2.94, 8.41)
Omnibus *P*-values	*P* < 0.0001			*P* < 0.0001				

Estimates shown are multinomial odds ratios complete with 95% confidence intervals. Estimates are accompanied by omnibus *P*-values derived from Wald tests (with 2 degrees of freedom).

a Estimates adjusted for the risk factors shown in Table 2.

### Attributable risk

Table 4 shows estimates of population-attributable risk for selected risk factors. Approximately 25% of risk of cannabis problems appear attributable to early-onset persistent conduct problems. Maternal smoking has a similar attributable risk, and the attributable risks of maternal cannabis use, early material disadvantage (manual parental social class, subsidized housing) and maternal postnatal depressive symptoms are also substantial.

**Table 4:** Population-attributable risk fractions (PARF) for problematic cannabis use (compared with no use/non-problematic use) for imputation sample of 7218

Predictor	Reference category for predictor	Risk category for predictor	PARF
Housing tenure	Mortgaged/owned/private rented	Subsidized rented	13%
Maternal education	A level or higher/O-level	< O-level	7%
Social class	Managerial/professional	III or lower	16%
Maternal cannabis	No	Some use postnatally	17%
Maternal alcohol	Less than daily	Daily use	3%
Maternal smoking	No	Some use postnatally	24%
Postnatal maternal depressive symptoms	Subthreshold throughout	EPDS > 12 at some point	15%
Conduct problems	Low/AO/CL	EOP	25%

EPDS = Edinburgh Postnatal Depression Scale; EOP = early onset persistent; AO = adolescent onset; CL = childhood limited.

## Discussion

In this large UK-based cohort of 16-year-olds recent cannabis use, without apparent problems, was relatively common—slightly more so among girls than among boys. A smaller proportion (fewer than 5%) of the cohort reported cannabis problems and these were more common among boys compared to girls. Cannabis problems, compared to cannabis use without apparent problems, were associated more strongly with risk factors in earlier life, most notably social disadvantage, maternal substance use, maternal depression and early-onset persistent conduct problems. Almost a quarter of cannabis problems at age 16 appeared possibly attributable to early-onset persistent conduct problems and almost a fifth possibly attributable to maternal tobacco and cannabis use. While the effects of early-life factors were attenuated in models that also considered conduct problems (and vice versa), these effects remained strong, possibly reflecting independent influences. This evidence is the first of its kind from the United Kingdom, complementing previous studies in North America, Australasia and northern Europe 4–31.

### Limitations

ALSPAC is subject to attrition, which is higher among the socially disadvantaged and increases with participant age. This may have implications for external validity, as we have discussed previously 10. It is also possible that we underestimated the prevalence of problem cannabis use as this outcome was associated with earlier exposures that, in general, were more common among the disadvantaged. Multiple imputation of missing data made little difference to either our estimates of the prevalence of cannabis use or problem use at age 16 or to our estimates of the effect of earlier exposures on these outcomes (full data for comparison available as a web supplement). There are few available UK data with which to compare our estimates. In the 2010/11 sweep of the British Crime Survey, 22% of male compared to 13% of female respondents aged 16–24 years reported any use of cannabis in the past year 32. For daily or almost daily use the equivalent proportions were 13 and 8% in this age group. In a recent large UK school-based study, 22% of 15 year-old boys and 20% of 15-year-old girls reported any use of cannabis in the previous year 33.

Most of our measures were self-reported by mothers or their children, which could introduce bias 34. Bias is most likely when both exposure and outcome are reported by the same individual, which was not the case in our study. We used a validated questionnaire instrument to distinguish between problem cannabis use and use without problems. The usual scoring system of the CAST questionnaire employs a higher cut-off score than we used to ascribe problem use. Our choice of the lower cut-off reflected both considerations around the age of our sample and around our patterns of response. Had we used a higher cut-off larger effects might have been apparent, but lower power would have led to these being estimated more imprecisely. The most probable outcome of misclassification in our measures arising through the mechanisms discussed above is a dilution of the apparent effects we estimated. We considered early-life exposures measured prior to measurement of conduct problems in an attempt to address issues of reverse causation. It is possible that some of these exposures, for example maternal substance use, persisted beyond the early life-course. Because of this, any inference on specific effects of early-life exposure on later outcomes must be cautious. Our sample was aged mainly 16 years at the time they completed our questionnaire. Problem cannabis use phenotypes might not have emerged fully by this point of the life-course and we might have identified risk factors for cannabis problems apparent relatively early in the life-course rather than cannabis problems in general.

Conduct disorder trajectories were measured between the ages of 7 and 13 years. Cannabis use in ALSPAC was first reported at age 10, when one participant reported cannabis initiation 35. A small proportion of participants (1.8%) reported initiation of cannabis use by age 13. Among these participants, it is possible that cannabis use exerted some influence on their later measures of conduct problems.

We are unable to make any strong statements about mediation, as we did not test this formally, and in any case our data are observational. The association between the early-life factors we studied (measured prior to measurement of conduct problems) and adolescent cannabis use was attenuated on adjustment for conduct problem trajectory. Similarly, the association between conduct problem trajectory and subsequent cannabis use was attenuated on adjustment for early-life factors. In most instances this attenuation was not substantial and effects remained strong. This provides moderate evidence that while some of the effect of early-life adversity on subsequent risk of cannabis use could be mediated through conduct problems, an effect independent of this pathway also appears to be operating. Similarly, conduct problems appear to increase the risk of subsequent cannabis use irrespective of whether or not they are preceded by adversity in earlier life. Any causal inference based on these estimates is necessarily cautious, given the difficulty of identifying independent effects of closely correlated covariates and given plausible levels of measurement error and categorical outcome variables 36.

### Comparison with other evidence

The prevalence of cannabis use we found is broadly similar to that seen in other studies. We are aware of one previous study reporting higher cannabis use among girls compared to boys 31. A consistent finding among our respondents was of higher problem cannabis use among boys. We confirmed the general association between conduct problems and cannabis use found in previous studies, adding evidence of specific nuances of this relationship 4–30. Childhood-limited conduct problems did not appear to be an important risk factor for later cannabis use. Adolescent-onset conduct problems appeared to be more of a risk factor for cannabis use rather than problem use. Early-onset persistent conduct problems, in contrast, appeared to be an important risk factor for problem cannabis use rather than any use.

### Potential mechanisms underlying the link between early-life factors, conduct problems and cannabis use

Our study is limited in its capacity to test mechanisms, as discussed above. Early-onset persistent conduct problems might increase the risk of adverse adolescent outcomes through fostering relations with more deviant peers, alongside alienation from more conventional institutions and behavioural pathways. Maternal substance use could influence offspring substance use through the messages it conveys around the acceptability of certain behaviours. Shared genetic factors might also be important. Twin studies consistently suggest high heritability of cannabis use, although important specific genetic influences remain unidentified 37,38.

## Conclusions

Adolescent cannabis use and problem use appear to have distinct multi-factorial causes. Problem cannabis use might be a later manifestation of a pattern of conduct problems established in early childhood, although it is also associated with disadvantage and maternal substance use, irrespective of whether or not these precede overt conduct problems. These associations could suggest a preventive strategy focused on these risk factors. Cannabis use without apparent problems is both much more common and apparently much less influenced by any risk factors studied. A risk factor-based approach to prevention of unproblematic cannabis use seems less appropriate.

### Declaration of interests

None.

## APPENDIX

### Correcting for classification errors

As stated in the Methods section, a ‘three-step’ approach 40 was chosen for the current analysis. For the first step, latent class growth analysis was used with Mplus to derive the trajectories of conduct problems, as described previously 10. Secondly, the posterior probabilities from this model were used to assign each respondent to the class for which this probability was greatest (known as modal class assignment). Thirdly, estimates from models involving the conduct trajectory measure were corrected for misclassification.

It has been shown recently that estimating the effect of covariates on a latent class measure can bias the parameter estimates unless a one-stage model is performed, in which covariate effects are estimated at the same time as the latent class measurement 40. However, Vermunt has described a number of instances in which the one-stage model is not ideal, and has proposed a method which uses the classification errors from the original mixture model to adjust for the bias within the subsequent regression analyses. This approach is very similar in most respects to the three-step method utilized regularly by trajectory modellers—the one difference being that uncertainty in the estimation of the latent class measure is incorporated into the multinomial regression model but, importantly, in a manner that introduces less bias than simply weighting by the posterior probabilities of trajectory membership. In Vermunt's method, the level of agreement between the underlying latent class measure and its predicted (manifested) counterpart forms a set of cell-weights which correct the parameter estimates for the bias mentioned above. This classification-error matrix can be calculated for any second-stage class-assignment procedure (e.g. modal class assignment, proportional assignment, random assignment) and furthermore, in the case of modal class assignment, the required matrix can be derived easily from that given in Mplus' standard output for a mixture model.

## Supporting information

Table S1 Characteristics of study sample according to availability of exposure and outcome data.
