# Supplementary material for: Childhood conduct disorder trajectories, prior risk factors and cannabis use at age 16: birth cohort study
Source: Addiction. 2013 Jul 12;108(12):2129–38. doi: 10.1111/add.12268 (PMC3908358; doi:10.1111/add.12268)
Supplement: Table S1 — Characteristics of study sample according to availability of exposure and outcome data. [file add0108-2129-sd1.docx]

Web table 1. Characteristics of study sample according to availability of exposure and outcome data

|  |  |  | Data availability | | |  |
| --- | --- | --- | --- | --- | --- | --- |
|  |  | n | No CP data | CP data but no cannabis information | CP data and cannabis information | X2, p |
| Gender | Male | 7220 | 3583 (53.0%) | 1871 (61.2%) | 1766 (42.5%) | X2 = 256.5,  p < 0.001 |
|  | Female | 6756 | 3175 (47.0%) | 1188 (38.8%) | 2393 (57.5%) |  |
| Housing tenure | Mortgaged/owned | 9559 | 3674 (61.6%) | 2359 (79.0%) | 3526 (86.5%) | X2= 881.4,  p < 0.001 |
|  | Private rented | 1384 | 825 (13.8%) | 252 (8.5%) | 307 (7.5%) |  |
|  | Subsidized rented | 2082 | 1465 (24.6%) | 373 (12.5%) | 244 (6.0%) |  |
| Parity | First born | 5770 | 2475 (42.0%) | 1275 (43.1%) | 2020 (49.5%) | X2= 111.2,  p < 0.001 |
|  | Second born | 4539 | 2028 (34.4%) | 1087 (36.8%) | 1424 (34.9%) |  |
|  | Third born plus | 2618 | 1388 (23.6%) | 594 (20.1%) | 636 (15.6%) |  |
| Home overcrowding | ≤1 person/room | 11924 | 5200 (89.4%) | 2805 (95.2%) | 3919 (97.0) | X2 = 242.2, p < 0.001 |
|  | >1 person/room | 878 | 616 (10.6%) | 143 (4.8%) | 119 (3.0%) |  |
| Maternal education | A level or higher | 4392 | 1322 (24.7%) | 1018 (34.3%) | 2052 (50.1%) | X2= 939.1,  p < 0.001 |
|  | O-level | 4296 | 1789 (33.5%) | 1144 (38.6%) | 1363 (33.2%) |  |
|  | < O-level | 3728 | 2238 (41.8%) | 805 (27.1%) | 685 (16.7%) |  |
| Household income | Top 20% | 1992 | 932 (29.5%) | 509 (17.9%) | 1052 (26.8%) | X2= 402.9,  p < 0.001 |
|  | Middle 60% | 5937 | 1782 (56.3%) | 1781 (62.5%) | 2374 (60.4%) |  |
|  | Lowest 20% | 2010 | 449 (14.2%) | 559 (19.6%) | 501 (12.8%) |  |
| Social class | Professional/managerial & technical | 6339 | 2183 (46.1%) | 1530 (54.6%) | 2626 (66.4%) | X2= 359.1,  p < 0.001 |
|  | Skilled non-manual or lower | 5162 | 2556 (53.9%) | 1274 (45.4%) | 1332 (33.7%) |  |
| Marital status at enrolment | Married (incl. divorced/widowed) | 10586 | 4404 (73.7%) | 2556 (85.3%) | 3626 (88.2%) | X2= 383.8,  p < 0.001 |
|  | Unmarried | 2499 | 1575 (26.3%) | 441 (14.7%) | 483 (11.8%) |  |
| Maternal age at delivery | < 25 years | 3337 | 2274 (33.7%) | 546 (17.9%) | 517 (12.4%) | X2= 864.4, p < 0.001 |
|  | 25-29 | 5403 | 2551 (37.8%) | 1260 (41.2%) | 1592 (38.3%) |  |
|  | 30-34 | 3850 | 1432 (21.2%) | 943 (30.8%) | 1475 (35.5%) |  |
|  | 35+ | 1386 | 501 (7.4%) | 310 (10.1%) | 575 (13.8%) |  |
